# An Inhibitor of the δPKC Interaction with the d Subunit of F_1_Fo ATP Synthase Reduces Cardiac Troponin I Release from Ischemic Rat Hearts: Utility of a Novel Ammonium Sulfate Precipitation Technique

**DOI:** 10.1371/journal.pone.0070580

**Published:** 2013-08-01

**Authors:** Mourad Ogbi, Ijeoma Obi, John A. Johnson

**Affiliations:** Department of Pharmacology & Toxicology and Program in Regenerative Medicine, Institute of Molecular Medicine and Genetics, Georgia Regents University, Augusta, Georgia, United States of America; University of Otago, New Zealand

## Abstract

We have previously reported protection against hypoxic injury by a cell-permeable, mitochondrially-targeted δPKC-d subunit of F_1_Fo ATPase (dF_1_Fo) interaction inhibitor [NH_2_-YGRKKRRQRRRMLA TRALSLIGKRAISTSVCAGRKLALKTIDWVSFDYKDDDDK-COOH] in neonatal cardiac myo-cytes. In the present work we demonstrate the partitioning of this peptide to the inner membrane and matrix of mitochondria when it is perfused into isolated rat hearts. We also used ammonium sulfate ((NH_4_)_2_SO_4_) and chloroform/methanol precipitation of heart effluents to demonstrate reduced card-iac troponin I (cTnI) release from ischemic rat hearts perfused with this inhibitor. 50% (NH_4_)_2_SO_4_ saturation of perfusates collected from Langendorff rat heart preparations optimally precipitated cTnI, allowing its detection in Western blots. In hearts receiving 20 min of ischemia followed by 30, or 60 min of reperfusion, the Mean±S.E. (n = 5) percentage of maximal cTnI release was 30±7 and 60±17, respectively, with additional cTnI release occurring after 150 min of reperfusion. Perfusion of hearts with the δPKC-dF_1_Fo interaction inhibitor, prior to 20 min of ischemia and 60–150 min of reperfusion, reduced cTnI release by 80%. Additionally, we found that when soybean trypsin inhibitor (SBTI), was added to rat heart effluents, it could also be precipitated using (NH_4_)_2_SO_4_ and detected in western blots. This provided a convenient method for normalizing protein recoveries between groups. Our results support the further development of the δPKC-dF_1_Fo inhibitor as a potential therapeutic for combating cardiac ischemic injury. In addition, we have developed an improved method for the detection of cTnI release from perfused rat hearts.

## Introduction

Cardiac ischemia/reperfusion (IR) injury is responsible for more annual deaths than any other medical condition. It occurs as a consequence of a heart attack when one or more of the coronary arteries become occluded, typically by atherosclerosis or thrombosis. This leads to regional deprivation of the myocardium to oxygen, nutrients and other factors, and if the blockage is not relieved promptly, cardiac cells experience severe energy deprivation, the loss of ion homeostasis, excessive proteolysis, oxidative stress, and additional damaging events, which culminate in cell death [1], [2]. Currently, the most successful strategy for minimizing cardiac IR injury is to restore blood flow and “reperfuse” the tissue. However, considerable injury also occurs during reperfusion [1], [2] and improved therapeutics for IR injury are desperately needed.

Following a prolonged IR insult, intracellular proteins are released from cardiac muscle cells and can be detected in serum as biochemical markers of cardiac IR injury [1], [3]. At present the preferred protein used for this purpose is cardiac troponin I (cTnI) [4]–[6]. cTnI is a myofibrillar protein which complexes with tropomyosin and troponins T and C to minimize Ca^++^ binding to troponin C, and prevent subsequent crossbridge formation between the actin and myosin myofilaments [7]. Hence, it plays a crucial role in the diastolic relaxation. It has only ∼50% amino acid homology to skeletal muscle TnI and is found almost exclusively in the heart [3]. The effects of cardiac IR injury on cTnI depend on the duration of the insult. During mild cardiac ischemia proteolysis of cTnI may not lead to its release from cells, and instead contributes to reversible contractile dysfunction known as myocardial stunning [8]. In prolonged ischemia or IR, cTnI is released into serum, mostly as a consequence of cardiac myocyte necrotic cell death [1]–[3].

Many laboratories have previously focused on mechanisms of cardiac IR injury and protection mediated by the protein kinase C (PKC) family [9]–[17]. In support of this, previous studies have reported a PKCε-selective activating peptide and a PKCδ translocation inhibitor peptide that induce cardio-protection from IR injury in cardiac myocytes [18], isolated hearts and *in vivo*, in transgenic mice [11], [17], [18]. Furthermore, a PKCδ-selective translocation inhibitor, administered immediately following a prolonged ischemic insult, improved the recovery of mouse heart ATP levels during oxygenated reperfusion [18]. We have also previously demonstrated, in neonatal cardiac myocytes, that the εPKC isozyme protects cytochrome oxidase [19]–[22] during PC and the δPKC isozyme interacts with the “d” subunit of F_1_Fo ATP synthase (dF_1_Fo) to inhibit F_1_Fo function, impair energetics, and reduce survival following prolonged hypoxia [14]–[16]. Instrumental in this latter work was a peptide developed in our laboratory, which inhibits the δPKC-dF_1_Fo interaction [15], [16].

The F_1_Fo-ATP synthase is the terminal complex in oxidative phosphorylation and supplies over 90% of cardiac energy, making it a logical focus of cardiac IR research. It is a 16-subunit enzyme complex associated with the inner mitochondrial membrane (IMM) [23]. During severe heart attacks the IMM gradient, which supplies the energy for F_1_Fo-ATP synthase, is lost [23]. As a consequence, the F_1_Fo-ATP synthase first becomes inhibited and then operates in reverse as an ATPase [1], [2], [23]. Initially this allows the F_1_Fo complex to pump protons out of the mitochondrial matrix which in-turn, re-establishes some of the lost IMM potential (Ψm) [23]. However, if ischemia is not interrupted, F_1_Fo ATPase contributes heavily to the loss of myocardial ATP and consequent cell death [1], [2], [23]. In addition, the return of aerobic ATP synthesis is impaired following heart attacks, during reperfusion, but the mechanisms for this delay are not completely known. Previous studies have proposed that cardiac preconditioning (PC), a cardio-protective response against IR injury, inhibits F_1_Fo ATPase-mode activity [24], [25], which may contribute protection by reducing ATP hydrolysis after ischemic insults. There have also been reports that PC induces an improved recovery of cardiac ATP levels following prolonged IR injury [1], [2], [6]. The F_1_Fo complex therefore, has the potential to impact thousands of energy-requiring processes that contribute to normal cardiac functioning and IR injury.

In our studies with the δPKC-dF_1_Fo inhibitor we wanted to complement other methods of quantifying infarct size (e.g. triphenyltetrazolium chloride (TTC) staining techniques) with a confirmatory biochemical marker of myocardial injury, such as cTnI release. This study provides the first demonstration of reduced cTnI release from Langendorff hearts perfused with the δPKC-dF_1_Fo interaction inhibitor prior to ischemic exposures.

## Materials and Methods

### Ethics Statement

Extreme care was taken to avoid pain or duress to animals used in this study. Briefly, hearts were isolated from adult male Sprague-Dawley rats (350 g) under complete anesthesia (see below). All experimentation was approved by The Georgia Regents University Institutional Animal Care and Use Committee (approval #2009-156) and was in compliance with The Guide for the Care and Use of Laboratory Animals published by the United States National Institutes of Health.

### Materials

Unless otherwise specified all protease inhibitors and chemicals were from Sigma Chemical Co. Crystalline ammonium sulfate ((NH_4_)_2_SO_4_) was from MP Biomedical. Electron microscopy fixatives, resins and other supplies were from Electron Microscopy Sciences (Ft. Washington, PA).

### Langendorff Heart Preparations

Anesthesia was conducted by intraperitoneal injection of ketamine HCl (100 mg/kg) and xylazine (10 mg/kg) as previously described [19], [22]. A surgical incision to open the chest was made and heparin (285 units/kg) was administered via injection into the inferior vena cava. Hearts were rapidly excised and rinsed in oxygenated (95%O_2_, 5%CO_2_), chilled modified Kreb’s buffer (115 mM NaCl, 4 mM KCl, 1.1 mM MgSO_4_^.^7H_2_0, 1.3 mM KH_2_PO_4_, 24 mM NaHCO_3_, 1.0 mM CaCl_2_, 5.5 mM glucose, 50 µUnit/ml insulin, 0.1 mM sodium pyruvate, 0.5 mM taurine, pH 7.4) and attached by the aorta to a Langendorff perfusion apparatus. Retrograde-perfusion was conducted with 37°C modified Kreb’s buffer at constant pressure (75 mmHg) and a water-inflated, balloon-tipped catheter was inserted into the left ventricle (LV) through the left atrium. LV end diastolic pressure was adjusted to 5 mm Hg and the volume of the balloon was not subsequently changed. LV catheter pressure was detected using a pressure transducer. Cardiac parameters were calculated using AD Instruments PowerLab software. Hearts were paced at 300 bpm using a Grass SD stimulator except during global ischemia when pacing was stopped.

### Homogenization of Hearts

Rat hearts were isolated as described above. Cardiac ventricles were minced into ∼2 mm cubes, followed by homogenization in chilled homogenization buffer (20 mM tris-aminomethane, pH 7.4, 1 mM EDTA, 1 mM EGTA, 1% (v/v) Triton X-100, 10 µg/ml each of phenylmethylsulfonyl fluoride (PMSF), aprotinin, leupeptin) for 1 min using a motorized tissue homogenizer. Samples were then placed on ice for 1 min. This procedure was repeated 3 times. Next, samples were centrifuged at 600×g to pellet Triton-insoluble proteins. The Triton-extracted supernatant was used as a positive control for cTnI immunoreactivity in western blots and also to optimize cTnI (NH_4_)_2_SO_4_ and chloroform/methanol (Chl/MeOH) precipitations.

### Perfusion of Hearts with δPKC-dF_1_Fo Modulatory Peptides and Ischemia/reperfusion (IR) Protocols

Following a 20 min equilibration period, rat hearts were perfused for 1 hr with 50 nM concentrations of the cell-permeable, mitochondrial-targeted δPKC-dF_1_Fo inhibitor [NH_2_-YGRKKRR QRRRMLATRALSLIGKRAISTSVCAGRKLALKTIDWVSFDYKDDDDK-COOH] peptide or scrambled-sequence (inactive) control peptide [NH_2_-YGRKKRRQRRRMLATRALSLIGKRAISTSV CADKIGWAVLRTKSLFDYKDDDK-COOH] at 37°C in oxygenated, modified Kreb’s buffer. Details of peptide syntheses/purification have been provided elsewhere [15], [16]. Peptides were not washed out prior to ischemia and we did not supplement reperfusion buffers with peptides.

### (NH_4_)_2_SO_4_) Precipitation of Proteins in Cardiac Effluents

(NH_4_)_2_SO_4%_ saturation levels were obtained using the online *EnCor Biotechnology Ammonium Sulfate Calculator Program* (<http://www.encorbio.com/protocols/AM-SO4.htm>). Effluents (50 ml) from Langendorff-perfused hearts were collected at the indicated times, frozen in liquid nitrogen, and stored at −80°C until use in (NH_4_)_2_SO_4_ precipitations. Thawed effluents were then placed in a 100 ml beaker with a stir bar, and gently stirred in an ice bath. (NH_4_)_2_SO_4_ was added slowly (∼1.5 g every 3 min), and allowed to dissolve until final concentrations were achieved. Samples were stirred on ice for an additional 1 hr., and then centrifuged (4°C) at 20,000×g for 15 min in a Beckman JA-20 fixed-angle rotor. Supernatants were carefully removed, so as not to disrupt the protein pellet at the bottom of the tube, and were discarded. The inside of the centrifuge tubes were blotted dry with Kimwipes, taking care to avoid the bottom 3 cm where the protein pellets were. Next, proteins were resuspended in 180 µl of solublization buffer (SB) (10 mM Tris-HCl, pH 7.4, 2 µg/ml each of PMSF, leupeptin, and aprotinin) and transferred to a 2 ml microfuge tube, on ice. This step was repeated twice to rinse residual amounts of precipitated protein out of the centrifuge tube. Each 180 µl wash with SB was placed on ice in a separate 2 ml microfuge tube. Following washes with SB, the large Beckman centrifuge tubes used for the (NH_4_)_2_SO_4_ precipitation were further washed with 600 µl of room temperature (RT) methanol, which was then transferred to one of the microfuge tubes that contained the 180 µl of SB wash prepared in the previous steps. This methanol step was repeated 2 additional times with each 600 µl methanol volume being placed in one of the remaining microfuge tubes (with 180 µl of SB wash). During these (SB/methanol) steps, the bottom 3 cm of the large Beckman centrifuge tubes were meticulously washed using a pipettor on all sides, at least 5 times, to recover as much protein as possible. From this point on, all steps were conducted at RT. Samples in the microfuge tubes containing SB (180 µl) and methanol (600 µl) were vortexed and then 150 µl of chloroform was added, followed by vortexing. Next, 450 µl of deionized water was added to each tube, the tube was vortexed, and then subjected to centrifugation (12,000 rpm) for 5 min in a microfuge. The top phase of the resulting solution was discarded and 450 µl of methanol was added to each tube, followed by vortexing. A second 12,000 rpm centrifugation was conducted and the supernatant was discarded. The inner walls of the microfuge tube were blotted dry with Kimwipes, being careful not to touch the pellet at the bottom of the tube. At this stage large white pellets were observed which contained substantial amounts of residual (NH_4_)_2_SO_4_. It was therefore, necessary to re-dissolve the pellets in 150 µl of SB and then repeat the entire Chl/MeOH precipitation protocol (as described above) one time to rid the sample of excess (NH_4_)_2_SO_4_. After the second Chl/MeOH extraction, the microfuge tube walls were blotted dry and the resulting pellets were dissolved in 40 µl of 1: 3 diluted Laemlli sample buffer, heated at 85°C for 5 min, and either stored at −20°C until use, or loaded directly on SDSPAGE gels.

### SDS Polyacrylamide Gel Electrophoresis and Western Blot Analyses

SDSPAGE (12% acrylamide gels) and electro-elution of proteins onto nitrocellulose paper (NCP) were as previously described [14], [16], [19], [22]. The resulting blots were stained with 0.05% (w/v) Ponceau S stain in 5% acetic acid to visualize proteins, and then probed with anti-cTnI (Santa Cruz (Cat. No sc-52277) or anti-FLAG (Sigma), each at 1∶500 dilution) or anti-SBTI (1∶30,000, Thermo Scientific) antisera. Detection was by horse radish peroxidase-coupled rabbit anti-mouse 1∶1500 (for anti-cTnI and anti-FLAG) or goat anti-rabbit 1∶1500 (for anti-SBTI)) secondary antisera, and enhanced chemiluminescence (GE Healthcare) [14]–[16].

### Isolation of Crude Mitochondrial Matrix and Inner Membrane Fractions for Western Blot Analyses

The δPKC-dF_1_Fo interaction inhibitor (50 nM) was perfused into Langendorff rat heart preparations for 1 hr at 37°C. Mitochondria were then isolated from hearts using Percoll/Optiprep density gradients [14]–[16], [19], [22] and subjected to sub-fractionation as previously described [26]. Briefly, 0.5 mg of isolated mitochondria were incubated 15 min on ice in 500 µl of hypotonic buffer (5 mM Tris HCl, 1 mM EDTA, pH 7.4), and then centrifuged at 20,000×g (10 min, 4°C). The resulting pellet, containing mitoplasts, was resuspended in 500 µl hypotonic buffer and sonicated on ice to rupture the inner mitochondrial membrane (IMM). After sonication the fragmented mitoplasts were subjected to centrifugation at 100,000×g. The pellet was used as the IMM-enriched fraction and the supernatant contained the mitochondrial matrix protein fraction. The IMM, and matrix fractions were precipitated using chloroform/methanol techniques and then solublized in Laemmil sample buffer. These samples were heated for 5 min at 85°C and used in western blots.

### Electron Microscopy

Fixation, embedding and sectioning of cardiac ventricle or isolated mitochondria and immunogold localization of the FLAG epitope-tagged δPKC-dF_1_Fo interaction inhibitor was performed by The Georgia Regents University Electron Microscopy Core Facility. For these experiments rat hearts were perfused in Langendorff mode for 1 hr, as described above, with 50 nM concentrations of the δPKC-dF_1_Fo interaction inhibitor. Hearts were then quickly removed from the Langendorff apparatus and either homogenized to prepare isolated mitochondria, or perfused with IEM fixative (see below) and 4 thick cross sections of the ventricles were prepared. One or more 2 mm cubes were rapidly excised from one of the sections. Tissue or mitochondria were fixed in 4% formaldehyde, 0.2% glutaraldehyde, 2 mM CaCl_2_, 4 mM MgSO_4_ and 0.1 mM sodium cacodylate overnight at 10°C. Samples (mitochondrial pellet or cubes from the left ventricle) were then washed in 0.1 M cacodylate buffer, followed by deionized water and were then dehydrated in a graded ethanol series up to 95% and embedded in LR White resin as previously described [27]. Thin sections were cut on a Leica EM UC6 ultramicrotome (Leica Microsystems, Inc. Bannock, IL) and collected on 200 mesh nickel grids. Sections were incubated for 2 hrs at room temperature in a humid chamber with blocking buffer (BSA (5%), normal donkey serum (3%), Tween (0.05%) in Tris-Buffered saline) prior to an overnight incubation at 4°C with M2 anti-FLAG antisera (Sigma) diluted in blocking buffer. Sections were then washed 4× at RT with blocking buffer and incubated with anti-mouse 15 nm circular gold particle-labeled secondary antibody for 2 hrs as previously described [28], [29]. Grids were washed twice with blocking buffer, followed by deionized water, then post-stained with 2% aqueous uranyl acetate and bismuth subnitrate. Samples were visualized using a JEM 1230 transmission electron microscope (JEOL USA Inc., Peabody, MA) at 110 kV and imaged with an UltrScan4000 CCD camera and First Light Digital Camera Controller (Gatan Inc., Pleasanton, CA).

### Statistical Analyses

Differences between two groups were assessed using unpaired Student’s t-test, and multiple group comparisons were evaluated using one-way ANOVA with Bonferroni’s post hoc test. A p value≤0.05 was considered significant.

## Results

### Chloroform/methanol (Chl/MeOH) Precipitation Improves Detection of Low Levels of Cardiac Troponin I in Western Blots

**Figure 1:**
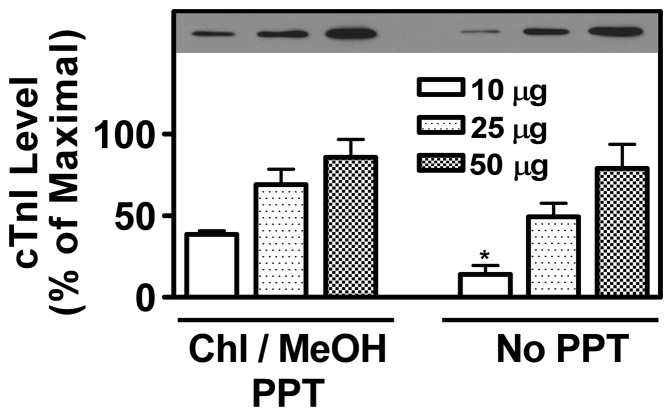
Chloroform methanol precipitation of proteins improves cardiac troponin I (cTnI) detection in Western blots. Cardiac left ventricular tissue from adult rats was homogenized as described in Methods. Homogenate protein (10–50 µg) was (left side) or was not (right side) subjected to chloroform/methanol precipitation prior to its resolution on SDSPAGE. Western blot analyses with anti-cardiac troponin I (cTnI) antisera were then conducted. A typical autoradiograph is shown (top). Histograms represent Mean±S.E. densitometry values, expressed as the percentage of maximal densitometry, from 4 independent experiments. Asterisk shown in the figure indicates statistically significant difference (p<0.01) between 10 µg protein groups for chloroform/methanol precipitated (left) vs. not chloroform/methanol precipitated (right) proteins.

As indicated in Methods, it was necessary to use Chl/MeOH precipitation of proteins following (NH_4_)_2_SO_4_ protocols to reduce the amount of residual (NH_4_)_2_SO_4_ in our samples prior to loading them on SDSPAGE gels for Western blots. We anticipated that the amount of cTnI released from our Langendorff heart preparations might be at the lower limit of detection and that there could be poor recovery of low amounts of cTnI following the Chl/MeOH extractions. We therefore, first evaluated the efficiency of Chl/MeOH precipitation of cTnI from rat heart homogenates (Figure 1). In these analyses, 10–50 µg of rat heart ventricle protein was subjected to either Chl/MeOH extraction prior to solublization in Laemmli sample buffer (Figure 1, left side) or was directly solublized in Laemmli sample buffer without Chl/MeOH extraction (Figure 1, right side). Samples were then subjected to SDSPAGE and western blot analysis using anti-cTnI antisera and enhanced chemiluminescence detection methods. In these analyses we detected a single cTnI species of ∼25 kDa, which is similar to the previously published molecular mass (26 kDa) for the rat holo-cTnI protein [30]. Of most importance, we observed no loss of cTnI detection when samples were precipitated with Chl/MeOH just prior to SDSPAGE. In fact, with 10 µg of cardiac ventricle protein there was a 64±14% improvement (Mean±S.E., n = 4, p<0.02 ) in cTnI detection (Figure 1).

### Utility of Exogenously Added Soybean Trypsin Inhibitor (SBTI) for Normalization of Protein Recoveries in (NH_4_)_2_SO_4_ cTnI Precipitations

**Figure 2:**
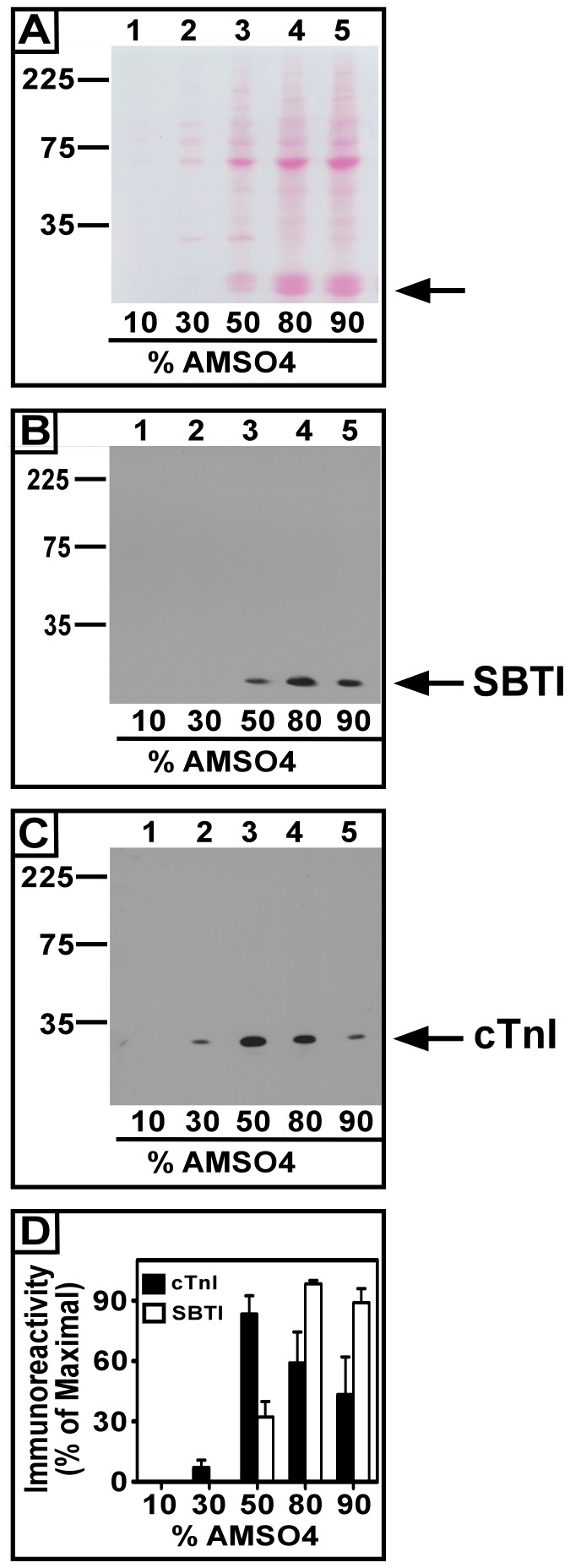
Optimization of ammonium sulfate (NH_4_)_2_SO_4_ precipitation of cardiac troponin I (cTnI) and the utility of soybean trypsin inhibitor as a normalization tool. In each experiment homogenate protein (200 µg) from rat cardiac left ventricle was added to each of 5 different aliquots of Kreb’s buffer (50 ml) on ice. The protease inhibitors leupeptin, aprotinin, phenylmethylsulfonyl-fluoride and soybean trypsin inhibitor (SBTI) were then added to each aliquot. Different amounts of (NH_4_)_2_SO_4_ were then added to each 50 ml sample to determine the optimal percentage of (NH_4_)_2_SO_4_ (0–90% (NH_4_)_2_SO_4_-saturated solutions) for precipitation of cTnI. Panel A is a Ponceau S. stain of a blot of the (NH_4_)_2_SO_4_ precipitated proteins from each sample. The arrow in Panel A marks the position of the most prominent protein band in the gel, which we later determined to be SBTI. Panel B is a Western blot for exogenously added SBTI. Panel C is a similar blot, but anti-cTnI immunoreactivity was monitored instead of SBTI immunoreactivity. Data in the histogram of Panel D shows Mean±S.E. autoradiograph densitometry scores, expressed as the percentage of maximal densitometry, from 5 independent experiments. Note the optimal level of (NH_4_)_2_SO_4_ saturation for the precipitation of SBTI was ≥80% whereas for cTnI it was 50%.

**Figure 3:**
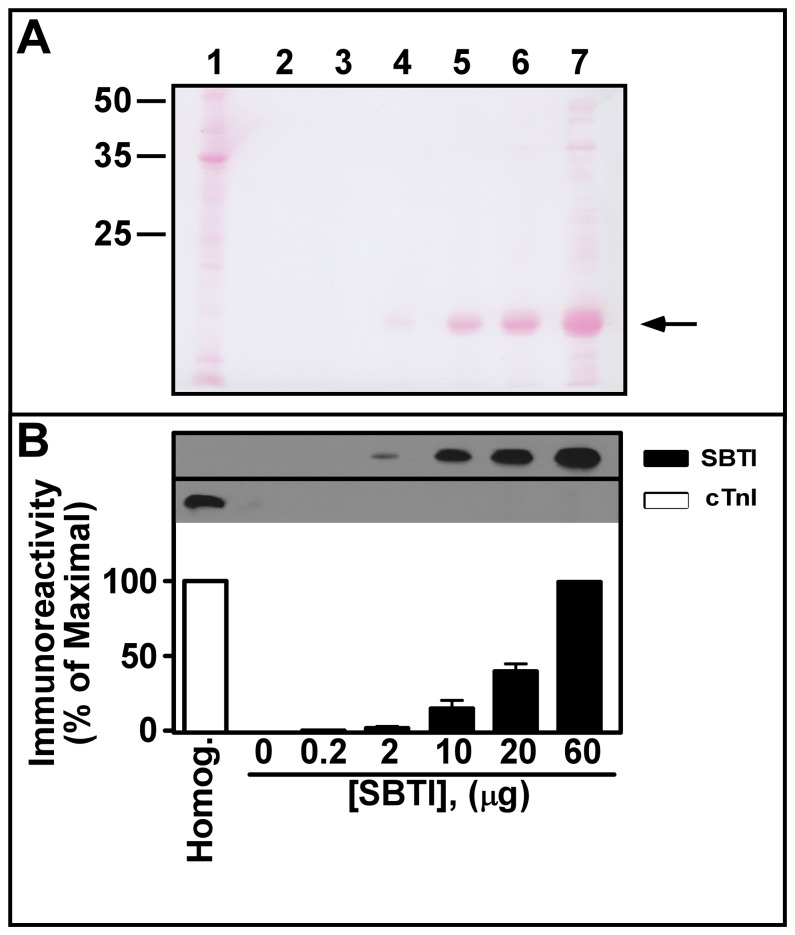
Commercially available soybean trypsin inhibitor (SBTI) preparations are devoid of cardiac troponin I (cTnI). Lane 1 contains 35 µg of rat left ventricle homogenate prepared as in Figure 1. Lanes 2–7 contain 0–60 µg of commercially available SBTI. All samples were subjected to SDSPAGE and proteins were electro-transferred onto nitrocellulose paper (NCP). The resulting blots were stained for total proteins using Ponceau S stain (Panel A) and then subjected to Western blot analyses for the detection of SBTI and cTnI (Panel B). Note the absence of cTnI immunoreactivity in the commercially available SBTI samples. Typical autoradiographs are shown in the top of Panel B with the histogram representing Mean±S.E. densitometry values from all autoradiographs (expressed as the % of maximal densitometry scores) from 5 independent experiments.

**Figure 4:**
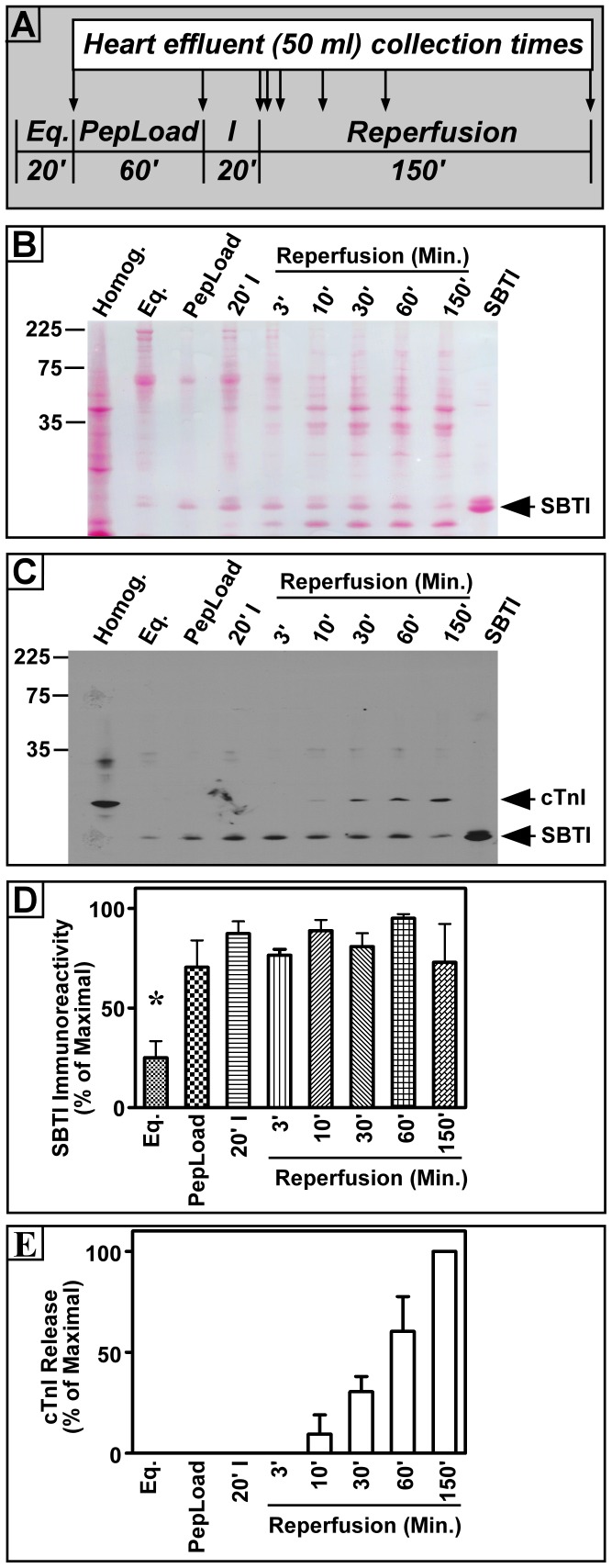
Detection of cardiac troponin I (cTnI) in effluents collected from Langendorff-perfused rat hearts following ischemia/reperfusion injury. Rat hearts were first given a 20 min equilibration (Eq) period, followed by a 1 hr perfusion (which will serve as the peptide loading phase (PepLoad), but no peptide was added for experiments in this figure). Next, hearts were given a 20 min global ischemia (I) exposure followed by 2.5 hr of perfusion with oxygenated Kreb’s buffer (Reperfusion). Effluents (50 ml) were collected from each of the protocol stages indicated by arrows in panel A. Proteins in each effluent were then precipitated by adding crystalline (NH_4_)_2_SO_4_ to a level of 50% saturation and centrifuging them. Precipitated proteins were subjected to SDSPAGE and transferred onto nitrocellulose paper (NCP). A representative Ponceau S stain of a typical NCP blot is shown in Panel B and autoradiographs from Western blots with anti-cTnI and anti-SBTI antisera are shown in Panel C. Mean±S.E densitometry values for SBTI (Panel D) and cTnI (Panel E) are also shown. cTnI densitometry values were normalized to SBTI levels in the same blots and SBTI values were not statistically different between groups in which cTnI was released. Results shown are typical of the responses of effluents collected from 5 independent rat hearts and are represented in the histograms.

In Figure 2, 50 ml of chilled Kreb’s buffer was supplemented with protease inhibitors (SBTI, leupeptin, aprotinin, PMSF), and 200 µg of rat heart ventricle homogenate (used as a source of cTnI). Proteins were then concentrated using (NH_4_)_2_SO_4_ precipitation and residual amounts of (NH_4_)_2_SO_4_ were removed using Chl/MeOH techniques. Maximal precipitation of total cardiac proteins occurred using solutions that were ≥80% saturated with (NH_4_)_2_SO_4_ (Figure 2A). Of interest, the major protein detected by Ponceau S staining in our nitrocellulose paper (NCP) blots was a protein of ∼20 kDa (See arrow, Figure 2A). This protein was not present in the rat heart homogenate (Figures 3 and 4, lane 1) loaded on gels as a positive control for cTnI immunoreactivity. This suggested to us that the unidentified ∼20 kDa protein was not likely coming from the cardiac ventricle protein in our samples. Since we did not add SBTI to the rat ventricle homogenate, but it was added to the 50 ml Kreb’s buffer samples used for cTnI precipitation we hypothesized that the ∼20 kDa protein might be SBTI. SBTI is not found naturally in rat hearts, and we added a known amount of it to each (NH_4_)_2_SO_4_ precipitated sample. We reasoned that SBTI might therefore, be useful as a standard to normalize the recovery of protein from the centrifugation steps in each (NH_4_)_2_SO_4_ precipitation. We next probed these blots with SBTI antisera in western blot analyses (Figure 2B). We consistently observed strong SBTI immunoreactivity in the same blot region as the Ponceau S stained ∼20 kDa protein. In our experiments SBTI did not precipitate with (NH_4_)_2_SO_4_ saturation levels at or below 30% saturation levels (Figure 2C–D). We found that 32+8% (X+S.E., (n = 6)) of SBTI maximal immunoreactivity was detectable in 50% (NH_4_)_2_SO_4_ saturated solutions (p<0.002 vs. 10% (NH_4_)_2_SO_4_), but maximal precipitation of SBTI (98+2% (p<0.0001 vs. 10% (NH_4_)_2_SO_4_), and 89+7% (p<0.0001 vs. 10% (NH_4_)_2_SO_4_), (Mean±S.E., n = 6)) occurred with samples that were 80 or 90% saturated with (NH_4_)_2_SO_4_ (Figure 2A and B). These results indicated that the combined (NH_4_)_2_SO_4_ and Chl/MeOH techniques we utilized, precipitated SBTI, allowing its reproducible detection it in western blots.

### Refinement of Protein Precipitation Techniques for Cardiac Troponin I (cTnI) Detection

We next conducted experiments in which 50 ml of Kreb’s buffer was supplemented with 200 µg of rat cardiac ventricular homogenate to determine the optimal (NH_4_)_2_SO_4_ solution saturation level for the precipitation of cTnI (Figure 2C). These analyses detected a single ∼25 kDa protein, which was maximally precipitated when solutions were ≥50% saturated with (NH_4_)_2_SO_4_ (Figures 2C–D). When compared to the 10% (NH_4_)_2_SO_4_ group we observed statistically significant increases in the cTnI precipitated in the 50% (p<0.0001), 80% (p<0.003), and 90% (p<0.04) (NH_4_)_2_SO_4_ groups. This is consistent with previous studies which used (NH_4_)_2_SO_4_ in the 30–60% saturation range to precipitate all troponin proteins from bovine heart or rabbit back and thigh muscles [31], [32]. However, these previous studies were not dealing with the low levels of cTnI found in our experiments, so we felt it was necessary to establish optimal conditions for our analyses. With 80% (NH_4_)_2_SO_4_ saturation, reproducible levels of cTnI were also detected, but they were 71% (n = 6, p<0.04) of the cTnI that was precipitated with 50% (NH_4_)_2_SO_4_. With 90% (NH_4_)_2_SO_4_ solutions we observed 52% of the 50% (NH_4_)_2_SO_4_ saturation level (Figure 2C–D). Recall that maximal SBTI precipitation was observed using (NH_4_)_2_SO_4_ saturation levels ≥80%. However, close analysis of the mean±S.E. data revealed that, even though SBTI precipitation was not maximal using 50% saturated (NH_4_)_2_SO_4_ solutions, a reproducible amount of SBTI was precipitated using either 50% or 80% saturated (NH_4_)_2_SO_4_ solutions. Also, solutions that were 50% saturated with (NH_4_)_2_SO_4_ contributed less residual (NH_4_)_2_SO_4_ in SDSPAGE analyses.

### Commercially Available SBTI is Free of Cardiac Troponin I (cTnI)

As a control we next confirmed that the SBTI used in these experiments (Sigma Chemical Co., #T9003) was devoid of cTnI. Figure 3 is a Western blot in which 0–60 µg of commercially available purified SBTI was detected using the above mentioned SBTI antisera. Lane 1 of Figure 3A represents 40 µg of rat cardiac ventricle homogenate. Note the absence of Ponceau S staining (Figure 3A) and immunoreactivity (Figure 3B) for the ∼20 kDa SBTI protein in this homogenate. In contrast, the homogenate yielded strong immunoreactivity for cTnI (Figure 3B, lane 1), but no cTnI was detected in samples containing only purified SBTI (Figure 3B, lanes 3–7). These results confirm the absence of cTnI in our SBTI preparations insuring that detectable cTnI in our experiments was not coming from the exogenously added SBTI.

### Exogenously Added SBTI can be used to Normalize (NH_4_)_2_SO_4_ Precipitations in Langendorff Heart Effluents Collected Following Ischemia/perfusion (IR)

The general treatment protocol for these experiments is shown in Figure 4A. Hearts were first given a 20 minute equilibration (Eq.) period to allow stable beating parameters to be achieved. Next, since the ultimate goal of these studies was to test the effects of our δPKC-dF_1_Fo interaction inhibitor peptide on cTnI release after IR injury, we subjected hearts to a 1 hr perfusion, which normally would reflect the peptide loading (PepLoad) phase. However, no peptide was given during this period in the experiments represented in Figure 4. After the PepLoad phase, a 20 min global, no-flow ischemia was administered to hearts followed by a 150 min oxygenated reperfusion period. We collected 50 ml aliquots of perfusate from the hearts at the end of each of these protocol phases and several additional 50 ml collections were made during the 150 min reperfusion period (See arrows in Figure 4A). Effluents were then subjected to (NH_4_)_2_SO_4_ precipitation (50% saturation level) and Western blot analyses for cTnI and SBTI as in Figure 2. Figure 4B shows a representative Ponceau S protein stain of the NCP used for the Western blot shown in Figure 4C. As expected, we observed some protein release immediately following the 20 min equilibration period, but very little protein was released after the 1 hr PepLoad period. In contrast, protein release increased immediately following the 20 min ischemia period, and with increasing reperfusion durations, we observed a time-dependent increase in total protein release, consistent with increasing cardiac cell injury (Figure 4B). Also of interest, we observed many more proteins being released in the reperfusion period than in any of the other stages of the protocol. Note that the SBTI recoveries observed in the Ponceau S stains (Figure 4B) were corroborated when we probed these same blots using the SBTI antisera (bottom, Figure 4C). Of interest, we observed significantly less SBTI precipitation in effluents collected from hearts immediately following the 20 min equilibration (Eq) period (Figure 4C and 4D) when compared to all other groups (Eq vs. 10 or 60 min p<0.001, Eq. vs all other groups p<0.05). The reason for this is currently unknown, but could reflect excessive proteases released from the hearts as they were recovering from excision from the animals and perfused *ex vivo*. Since we did not intend to compare cTnI release under this equilibration condition these findings had minimal impact on our study. Of most importance we observed no significant differences in the Mean±S.E SBTI levels precipitated by 50% saturated solutions of (NH_4_)_2_SO_4_ between any of the other groups (Figure 4D) indicating that SBTI precipitation was not altered by the ischemia/reperfusion manipulations of our protocol. We therefore, propose that SBTI can be used as a normalization tool, not only when cTnI in freshly harvested homogenate proteins are assayed (Figure 2), but also in effluents collected from Langendorff hearts that were subjected to IR (Figure 4C–D).

### (NH_4_)_2_SO_4_ Precipitation and Western Blot Analysis Reveal Progressive Release of cTnI from Perfused Rat Hearts

Figures 4C and E demonstrate significant detection of cTnI release from our Langendorff heart preparations following 20 min of ischemia and 30 min of reperfusion. We actually began to see modest cTnI release as early as 10 min of reperfusion in some experiments. The Mean±S.E. (n = 5) % of maximal cTnI release observed following 20 min of ischemia and 30, 60 or 150 min of reperfusion were determined to be 30±7 (p<0.005), 60±17 (p<0.008), and 100 (0.0001) %, respectively. Prior to developing this technique we had difficulty consistently estimating cardiac cell necrosis after IR using cTnI release with commercially available ELISAs, or by using triphenyltetrazolium chloride staining of heart sections, unless at least 90 min of reperfusion was given (not shown). This is an important point since the time course of increasing cTnI release, rather than levels at a single point, are often the most convincing evidence for confirming myocardial infarction in clinical situations [33]. Further, our results may suggest earlier events in the IR injury spectrum, which could be modulated by cardio-protective substances. Another interesting point with regard to the use of sample normalization with SBTI can be seen in the last (right most) effluent tested in Figure 4C. Note that there was substantial cTnI detected following 150 min of reperfusion in western blots. However, based on the low SBTI immunoreactivity corresponding to that gel lane, we hypothesize that there was poor protein recovery in that group in this experiment. Consequently, with SBTI normalization the amount of cTnI released in that group is higher than that shown in the blot. These results demonstrated the utility of this approach for monitoring cTnI release following IR injury in this model.

### Perfusion of Langendorff Rat Heart Preparations with the δPKC-dF_1_Fo Interaction Inhibitor Reduces cTnI Release Following Prolonged IR Injury

**Figure 5:**
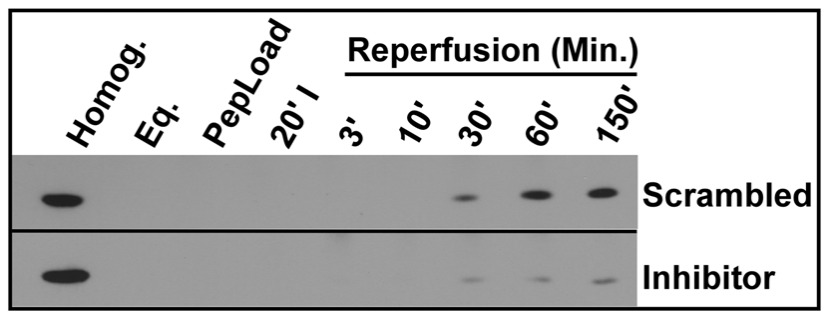
The δPKC-dF_1_Fo interaction inhibitor reduces cardiac troponin I (cTnI) release when perfused into Langendorff hearts. Experimental details were as described in the legend to Figure 4 except that during the peptide loading phase 50 nM concentrations of the scrambled sequence (inactive) control peptide or the δPKC-dF_1_Fo interaction inhibitor peptide were administered to hearts for 1 hr. Effluents were collected and Western blot analyses for cTnI were conducted. The left-most lane contains rat left ventricle homogenate used as a positive control for cTnI immunoreactivity. The remaining groups are described in the legend to Figure 4. Results shown are typical of the responses of 3 independent rat hearts in each peptide treatment group.

In Figure 5, hearts were perfused as in Figure 4A however, in this case, 50 nM concentrations of the cell-permeable, mitochondrially-targeted δPKC-dF_1_Fo interaction inhibitor, or a scrambled-amino acid sequence inactive control peptide [15], [16], were administered during the 1 hr PepLoad phase of the protocol. Each of these peptides contains an HIV-Tat protein transduction sequence [34] and a second mitochondrial targeting sequence [35]. Following peptide loading, hearts were exposed to 20 min of global ischemia and 150 min of oxygenated reperfusion. Samples of perfusate (50 ml) were collected at different stages of the IR protocol as shown in Figure 4A. Released proteins were concentrated using (NH_4_)_2_SO_4_ techniques (50% saturation) with SBTI normalization, and cTnI levels were monitored by Western blot. In the scrambled sequence control group we found the release of cTnI (Figure 5) to parallel that observed in hearts receiving no peptide (Figure 4). In contrast, hearts receiving the 1 hr preincubation with the δPKC-dF_1_Fo inhibitor peptide showed a reduction in normalized cTnI release at 60 and 150 min of reperfusion when compared to the scrambled sequence control peptide. On average the percent reductions of cTnI release by the δPKC-dF_1_Fo inhibitor at these time points were 80+7 (p<0.01, n = 4) and 69+17 (p<0.05, n = 4), respectively. These results indicate that this method can be used as a confirmatory marker of myocardial injury in Langendorff hearts subjected to IR. Further, it allows protection in Langendorff heart models to be detected at earlier reperfusion times than TTC staining or (in our experiments) cTnI ELISAs.

### The δPKC-dF_1_Fo Interaction Inhibitor Partitions to the Mitochondrial Inner Membrane (IMM) and Matrix when Perfused into Rat Hearts

**Figure 6:**
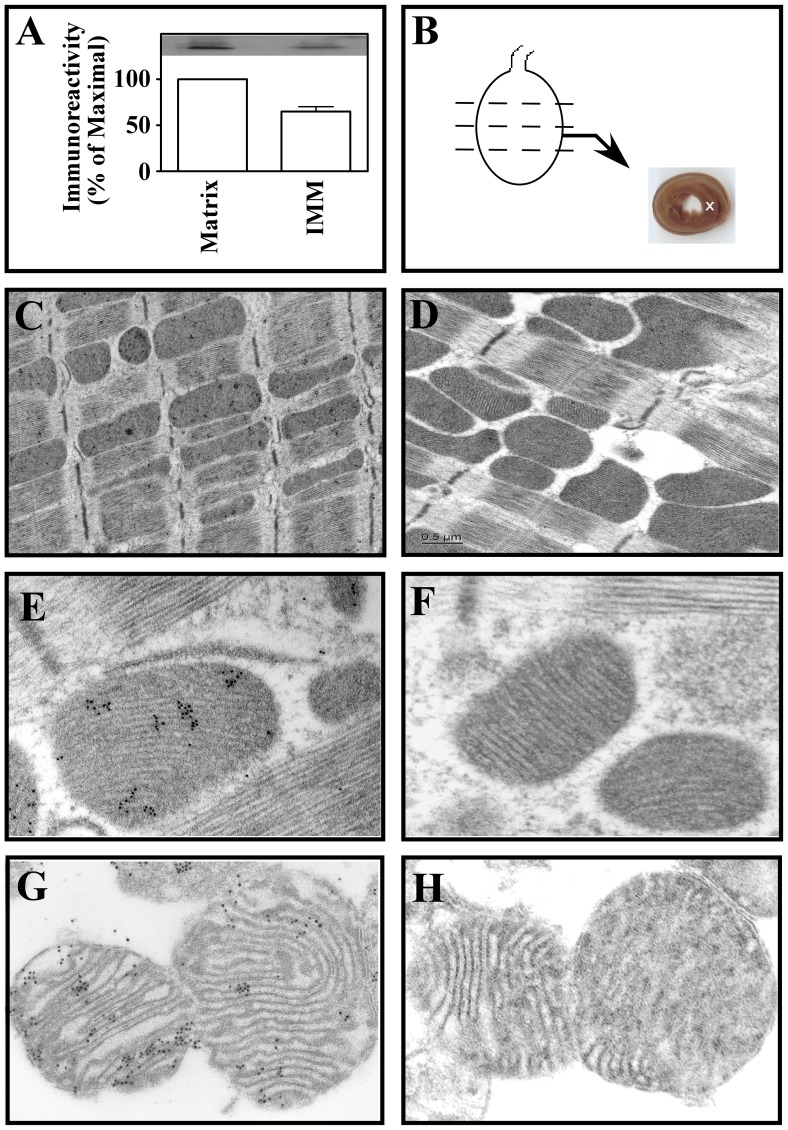
Sub-mitochondrial Localization of the δPKC-dF_1_Fo peptide inhibitor after its perfusion into Langendorff rat hearts. Adult rat hearts were first perfused with 50 nM concentrations of the FLAG-epitope tagged δPKC-dF_1_Fo inhibitor peptide for 1 hr. Hearts were then washed for 5 min with Krebs buffer that contained no peptide. In Panel A mitochondria were then isolated from the left ventricle and subfractionated into mitochondrial inner (IM) and matrix fractions as described in Methods. Sub-mitochondrial fractions were then probed in Western blots with monoclonal mouse anti-FLAG antisera at 1∶500 dilution. Results are expressed as the mean±S.E. percentage of the maximal densitometry scores from 3 independent experiments. Extensive FLAG immunoreactivity was observed in the matrix and IMM fractions where δPKC would be predicted to interact with the “d: subunit of F1Fo ATP synthase. Panels B–H refer to results from immuno-gold transmission electron microscopy studies. Results shown are typical analyses conducted in 3 independent experiments in which random fields were selected from embedded sections and a total of 364 mitochondria were analyzed.

Our previous studies clearly demonstrated cardiac mitochondrial uptake of the δPKC-dF_1_Fo interaction inhibitor, but we did not provide evidence of its presence in the mitochondrial matrix or IMM where the F_1_Fo complex exists. We felt that demonstration its presence at these submitochondrial sites would strengthen our overall hypothesis that the δPKC-dF_1_Fo binding interaction contributes to cardiac ischemia reperfusion injury by inhibiting the F_1_Fo enzyme complex and that the δPKC-dF_1_Fo interaction inhibitor attenuates this interaction to protect against this injury. We therefore, conducted studies to determine if perfusion of rat hearts with this peptide allowed its targeting to these submitochondrial sites. In Figure 6 hearts were perfused in Langendorff mode with 50 nM concentrations of the FLAG epitope-tagged δPKC-dF_1_Fo inhibitor peptide for 1 hr at 37°C. Hearts were then perfused for 5 min with Krebs buffer without peptide and left ventricles were harvested for western blot analyses with anti-FLAG antisera. Figure 6A illustrates FLAG immunoreactivity in crude IMM, and mitochondrial matrix fractions isolated by hypotonic lysis and differential centrifugation techniques as described in Methods. Since the “d” subunit of F_1_Fo ATP synthase (dF_1_Fo) is found in the mitochondrial matrix our results suggest that following perfusion of hearts with the δPKC-dF_1_Fo inhibitor, sufficient FLAG epitope-tagged inhibitor peptide is delivered to the matrix to bind δPKC and prevent its interaction with dF_1_Fo. It is important to note that the protein concentration of the IMM fraction was 5 times greater than that of the matrix fractions. In the autoradiograph shown in Figure 6A we loaded all of the protein obtained for the matrix fractions from 0.5 mg of purified mitochondria. In contrast, only one-fifth of the total protein for the IMM in these analyses was loaded on gels to avoid protein overloading. Densitometry as shown in the Figure 6A was not corrected to reflect this. However, these data suggest the presence of our FLAG-tagged peptide inhibitor at the mitochondrial IMM and matrix where the F_1_Fo ATP synthase exists.

To complement our western blot studies we next conducted transmission electron microscopy (TEM) experiments on left ventricle Figure 6B–F) and mitochondria (Figure 6G and H) isolated from rat hearts perfused with 50 nM concentrations of the δPKC-dF_1_Fo inhibitor for 1 hr. In Figure 6C–H hearts were first perfused with this compound, perfused an additional 5 minutes with oxygenated Krebs buffer without the peptide, and then subsequently perfused with fixative (Methods). Cardiac ventricular tissue (as shown in Figure 6B) was then cut into 4 cross sections. From one of these sections (as shown by the white “X” in Figure 6B) we excised a 2 mm cube of tissue for embedding and the preparation of thin sections for TEM. Thin sections were blocked and probed with a 1∶5 dilution of mouse monoclonal anti-FLAG antisera and then subjected to immunogold labeling using gold particle-labeled anti-mouse secondary antisera. Figure 6C shows a representative cross section of cardiac ventricle from hearts loaded with the FLAG epitope-tagged δPKC-dF_1_Fo interaction inhibitor peptide. Note the substantial areas of high density (gold particle) staining in most mitochondria (Figure 6C) compared to the “no FLAG antisera control” (Figure 6 D). Figure 6E shows an enlargement of a mitochondria from a left ventricle section prepared from hearts loaded with our peptide. Note the substantial immuno-gold labeling observed in the mitochondrial cristae, and matrix and outer membrane and the scarcity of staining in extra-mitochondrial areas. As shown in Figures 6C, D and E we also observed some staining in the mitochondrial outer membrane. We hypothesize that the presence of the δPKC-dF_1_Fo inhibitor in the OM reflects its interaction with TIM/TOM mitochondrial protein uptake machinery as it is taken up into mitochondria and ultimately delivered to the IMM or matrix. In contrast, in thin sections prepared identically to those in Figure 6E we observed virtually no immunogold labeling when the anti-FLAG primary antisera (but not the gold-labeled secondary antisera) was omitted. Panels G and H of Figure 6 represent similar analyses to those in Figure 6E and F except density gradient-purified mitochondria, rather than left ventricle tissue, were fixed, sectioned and then probed for anti-FLAG immunoreactivity followed by gold particle-labeled secondary antibody. Similar to our results with anti-FLAG immunogold stained sections from intact ventricle, Figure 6G shows substantial labeling of the mitochondrial matrix, IMM, and OM. Figure 6H represents the control staining in purified mitochondria when the anti-FLAG antisera was omitted. Collectively these data represent the first demonstration that the δPKC-dF_1_Fo interaction inhibitor partitions to sub-mitochondrial sites when perfused into rat hearts. Of most importance, we observed substantial presence of the peptide in the mitochondrial matrix where the “d” subunit of F_1_Fo ATP synthase exists and hence the proposed site of the δPKC-dF_1_Fo interaction which we hypothesize inhibits F_1_Fo functions during cardiac IR. Our peptide therefore, appears to be delivered to the correct submitochondrial loci when perfused into Langendorff rat hearts to antagonize this interaction.

## Discussion

Considerable effort has gone into the development of rapid ELISA assays for the detection of cTnI released into the bloodstream as a confirmatory marker of myocardial infarction. cTnI can be quite stable in human blood with increases in its levels being readily detectable at 6–8 hr after ischemia and it can remain elevated for days [8]. Further, newer “high sensitivity cTnI assays may allow detection of “normal”, (non-MI) cTnI levels and also allow a more rapid detection of elevated cTnI levels in patients having an MI [4,33,36). Many recent studies have used ELISA detection of cTnI release from perfused hearts as an indicator of cardiac injury [37]–[41]. Therefore, the reasons for our lack of consistent cTnI detection using a high sensitivity rat cTnI ELISA are not fully understood. Many factors can alter cTnI detection in ELISA-based assays, some of which may help to explain our results. These include: poor cTnI recoveries during sample volume reduction or centrifugation steps; the use of different cTnI antisera in the ELISA kits [5], [8], cTnI proteolysis [5], [8], [30], [42]–[44]/phosphorylation [30], [32], [42]/complexing with other proteins [30], and the concentrations of fibrin or anticoagulants in samples [44]. In most cases, the results from different ELISA kits are not even directly comparable, with up to 20-fold variations in cTnI values being reported using different cTnI ELISAs on the same samples [42], [44]. There is also currently no cTnI standard to normalize the results from one cTnI ELISA to another [4], [36]. Also of importance, cTnI levels can be elevated under numerous non-myocardial infarction (MI) conditions such as in marathon runners [45], hypertensive emergencies [46] toxicity due to chemotherapeutic agents [47,48), and chest trauma [49]. Therefore, even though cTnI is currently the preferred confirmatory biochemical marker of MI, and is often very helpful in this context, obtaining consistent cTnI values, and the interpretation of cTnI ELISA results, can be complicated. For this reason, clinical cTnI levels are used in conjunction with other indicators of MI such as electrocardiograms and echocardiography. Finally, and of direct relevance to this study, different animal species have been reported to have variable responses in ELISA-based cTnI detection and cTnI can also be elevated in those models under non-ischemic stress conditions [50], [51].

Indeed, numerous sources of variability exist with current cTnI detection assays. Therefore, to confirm the cardio-protective actions of our δPKC-dF_1_Fo interaction inhibitor using a biochemical marker we found it necessary to use a non-ELISA-based approach. We first piloted concentration of 50 ml Langendorff effluents using Centriprep-10 concentrators and found very sporadic and often non-existent cTnI detection. This was not improved if Centriprep concentrated samples were subjected to chloroform-methanol precipitation. Since the concentration of these samples required long centrifugation periods and often the rate of sample concentration varied between samples, we believe that the time required for these concentrations may have allowed excessive proteolysis, oxidation or non-selective adherence of cTnI protein to the concentrator. Next, we attempted acid protein precipitation of rat heart homogenate protein (e.g. perchloric and others) which were also unsuccessful. Essentially, we observed normal protein band patterns in our (acid precipitation) blots by Ponceau S. staining, but we could not detect cTnI or SBTI in these Western blots. We experimented with a range of concentrations of total heart homogenate protein that was extracted with perchloric or other acids. We also neutralized homogenates to pH 7, and even performed a second precipitation step using chloroform/methanol. None of these manipulations allowed us to detect cTnI or SBTI in Western blots. It is possible that the acid extraction or one of the subsequent steps altered crucial epitopes in the cTnI or SBTI proteins such that our antisera could no longer detect them in Western blots. We then tested the (NH_4_)_2_SO_4_ protein precipitation method and found that it obviated the above mentioned problems encountered with other concentration methods.

Another important point is that *in vivo*, mostly cTnI fragments, not the holo-protein are found in serum after an MI. This has been found in many Langendorff heart studies as well. We found the release of only a ∼25 kDa cTnI protein species after ischemic injury (Figures 4–5). We therefore, cannot rule out the possibility that additional cTnI fragments were not detectable using our approach and therefore, the effects of the δPKC-dF_1_Fo inhibitor on the release or stability of these fragments is unknown. Numerous studies have reported cTnI proteolysis or other modifications, in cardiac myocytes or serum, following different periods of ischemia and reperfusion [8], [30], [42], [43]. For example, studies by McDonough et. al subjected rat hearts to 0, 15 or 60 min of ischemia followed by 45 min of reperfusion and applied a TnC affinity column, cTnI antisera and spectrometric analyses to demonstrate progressive degradation of cTnI as the intensity of ischemic insult was increased [30]. These authors found that rat holo-cTnI has 210 amino acids (AAs) and was progressively degraded to fragments containing AAs 1–193, 63–193 and 73–193, respectively. Degraded cTnI fragments also formed complexes with troponin C (TnC), and troponin T (TnT), some of which may occur because of crosslinks induced by transglutaminase (30). There were numerous methodological differences between their study and ours. For example, we did not use a TnC affinity column to purify cTnI. We observed a 25 kDa species of cTnI that was released from perfused rat hearts following IR injury and did not determine if this was holo-cTnI or a cTnI fragment, only that it was detected in western blots by an antisera directed at a cTnI epitope in the center of the protein. We selected this antisera because previous studies have suggested that, following IR injury, cTnI is progressively proteolyzed from the N- and C-termini and antisera directed towards an epitope in the center of the protein should detect most of these fragments. It is possible that the other fragments detected by McDonough et. al were generated in our experiments, but not released because the duration of ischemia used in our experiments was different, or we used different electrophoresis conditions.

In another study, Labugger et. al developed Western blot techniques to detect holo-cTnI and 11 fragments of cTnI in the serum of patients undergoing acute MI [42]. These patients however, did not have the precisely controlled durations of ischemia and reperfusion found in our experiments and insults were likely longer. These authors also spiked human serum with recombinant cTnI and incubated the serum with alkaline phosphatase. Their results indicated that much of the cTnI found in human serum from MI patients is phosphorylated or proteolyzed [42]. Additionally, one study reported the loss of at least some cTnI fragments in Langendorff perfused hearts after ischemia if reperfusion is conducted for >1 min [8], much shorter than the reperfusion points used in our study. In addition, cTnI has also been reported to be proteolyzed inside of cardiac myocytes after 15–25 min of global ischemia [8] or in ventricular biopsies from patients undergoing cardiac bypass surgery [43]. Further, the C-terminus of cTnI can be degraded when recombinant cTnI is exogenously added to human serum [44]. Also, covalent complexes between cTnI/cTnC and cTnI/cTnT have been reported after brief ischemia and may be lost with prolonged ischemia [30]. Finally, PKA-induced phosphorylation of cTnI at its N-terminus may prevent cTnI degradation [30]. It is therefore, possible that the ∼25 kDa cTnI species we detected was somewhat protected from degradation because it was either complexed with other proteins or phosphorylated.

Our previous studies demonstrated improved energetics and survival in neonatal cardiac myocytes pretreated with the δPKC-dF_1_Fo interaction inhibitor just prior to prolonged hypoxia [16]. In work to be published elsewhere we have also demonstrated that Langendorff hearts incubated with the δPKC-dF_1_Fo inhibitor enhanced ATP levels following a prolonged IR protocol. We propose that excessive inhibition of the F_1_Fo synthase by a δPKC interaction with the dF_1_Fo subunit plays a significant role in cardiac IR injury, and that our novel peptide could provide a mechanism for attenuating these effects. This study also demonstrates for the first time that our FLAG epitope-tagged δPKC-dF_1_Fo interaction inhibitor peptide could be detected at the mitochondrial matrix and IMM where the F_1_Fo ATP synthase complex exists. We have further shown the protective actions of our peptide against cardiac IR injury by demonstrating reduced release of the clinically relevant biochemical marker of cardiac cell necrosis, cTnI in hearts perfused with the δPKC-dF_1_Fo inhibitor. Regardless of whether we could detect all previously reported cTnI fragments, we did observe significant decreases in the release of the ∼25 kDa cTnI species when hearts were preincubated with the δPKC-dF_1_Fo inhibitor and not with a scrambled-sequence, inactive control peptide (Figure 5). This inhibitor also reduces infarct size when monitored using triphenyltetrazolium chloride staining of heart sections (not shown). These results corroborate one another and support a highly protective role for the δPKC-dF_1_Fo inhibitor against cardiac IR injury. The present study provides the first evidence for protection of intact rat hearts against IR injury by this energy-preserving compound and describes a novel approach for monitoring cTnI release from perfused rat hearts. Collectively, this work supports the development of a novel class of translational pharmaceutical to protect hearts against ischemic injury.
